# Mineral absorption is an enriched pathway in a brain region of restless legs syndrome patients with reduced *MEIS1* expression

**DOI:** 10.1371/journal.pone.0225186

**Published:** 2019-11-14

**Authors:** Faezeh Sarayloo, Alexandre Dionne-Laporte, Helene Catoire, Daniel Rochefort, Gabrielle Houle, Jay P. Ross, Fulya Akçimen, Rachel De Barros Oliveira, Gustavo Turecki, Patrick A. Dion, Guy A. Rouleau

**Affiliations:** 1 McGill University, Department of Human Genetics, Montréal, QC, Canada; 2 McGill University, Montreal Neurological Institute, Montréal, QC, Canada; 3 McGill University, Department of Psychiatry, McGill Group for Suicide Studies, Douglas Institute, Montréal, QC, Canada; 4 McGill University, Department of Neurology and Neurosurgery, Montréal, QC, Canada; Cinvestav, MEXICO

## Abstract

Restless legs syndrome is a common complex disorder with different genetic and environmental risk factors. Here we used human cell lines to conduct an RNA-Seq study and observed how the gene showing the most significant association with RLS, *MEIS1*, acts as a regulator of the expression of many other genes. Some of the genes affected by its expression level are linked to pathways previously reported to be associated with RLS. We found that in cells where *MEIS1* expression was either increased or prevented, mineral absorption is the principal dysregulated pathway. The mineral absorption pathway genes, *HMOX1* and *VDR* are involved in iron metabolism and response to vitamin D, respectively. This shows a strong functional link to the known RLS pathways. We observed the same enrichment of the mineral absorption pathway in postmortem brain tissues of RLS patients showing a reduced expression of *MEIS1*. The expression of genes encoding metallothioneins (MTs) was observed to be dysregulated across the RNA-Seq datasets generated from both human cells and tissues. MTs are highly relevant to RLS as they bind intracellular metals, protect against oxidative stress and interact with ferritins which manage iron level in the central nervous system. Overall, our study suggests that in a subset of RLS patients, the contribution of MEIS1 appears to be associated to its downstream regulation of genes that are more directly involved in pathways that are relevant to RLS. While MTs have been implicated in the pathogenesis of neurodegenerative diseases such as Parkinson’s diseases, this is a first report to propose that they have a role in RLS.

## Introduction

Restless legs syndrome (RLS) is a common sleep-related sensory-motor disorder with a high genetic predisposition. Twin studies have established the heritability of RLS to be approximately 50–60% [1–3]. Linkage studies and genome wide association studies (GWAS) have identified eight and 19 loci associated with RLS, respectively [4–12]. While the identification of loci offers valuable insights toward a better understanding of the pathogenicity, they collectively account for less than 10% of the heritability estimated for RLS [12, 13]. The biology of RLS is incompletely understood but the availability of iron and dopamine in the brain were reported to affect the severity of the sensory and motor symptoms in affected individuals [14, 15]. Moreover, kidney disorders, changes in circadian rhythm, multiple pregnancies as well as vitamin D deficiency were observed to correlate with RLS through unknown mechanisms [16, 17].

The strong association between RLS and *MEIS1* (p-value = 1.1E-180, odds ratio (OR) = 2.16 95% confidence interval (CI) 2.014–2.49), the regulatory role of MEIS1 over another RLS risk factor, *SKOR1*, (p-value = 1.09E-48, OR = 0.80 95%CI 0.77–0.83) [12, 18] and the homeobox transcription factor functionality of MEIS1, led us to hypothesize that MEIS1 might have downstream targets that would directly contribute to the pathogenesis. To better define the role of *MEIS1* and identify additional genes that may be involved in RLS, we generated and studied different transcriptomes (by RNA-Seq). The first set of transcriptomic data was derived from wild-type SK-N-SH cells (a neuronal-like human cell line [19]) and SK-N-SH cells stably overexpressing *MEIS1*. We hypothesized that genes which would be differentially expressed between these two lines could be MEIS1 regulated genes. In order to establish any link between these genes and RLS pathways, we postulated that their expression should also be altered in the brains of individuals who had RLS.

Following the analysis of the cell lines transcriptome data, we generated RNA-Seq data from postmortem tissues (ten thalamus and ten pons) obtained from RLS cases; this selection was based on our previous report highlighting the decreased levels of *MEIS1* mRNA and protein in cells and brain tissues of RLS cases who carry a *MEIS1*-risk haplotype (this reduced expression was only true for thalamus samples, nevertheless we also included pons to assess whether the previous observations will be replicated) [20]. In each thalamus and pons group, five individuals had significantly reduced *MEIS1* expression levels in comparison to the other five.

We find that mineral absorption is the only significantly altered pathway common in the cells and patients’ thalamus RNA-Seq datasets. Overall this study highlights the importance of the regulatory role of MEIS1 in RLS and suggests dysregulation of a new pathway.

## Results

### Characterization of *MEIS1* overexpression in SK-N-SH cells

**Fig 1:**
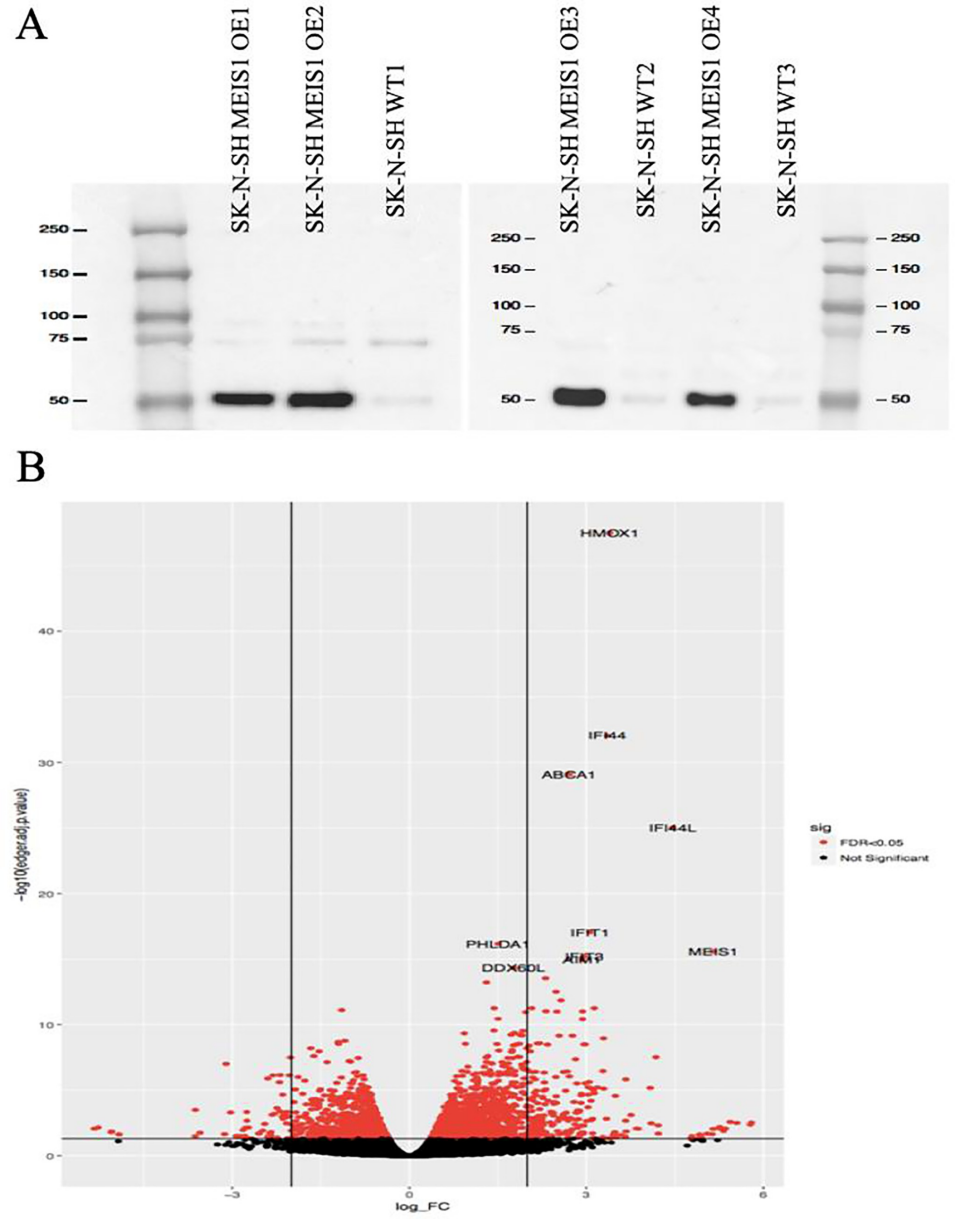
Characterization of *MEIS1* overexpression in SK-N-SH cells. **A.** Western blot analysis of *MEIS1*-OE cells. MEIS1 overexpressed in SK-N-SH cells migrates as a 50 kDa protein (Four clones were generated and only clones 1 to 3 were used for RNA-Seq, MEIS1 apparent molecular weight is 43 kDa). **B.** Volcano plot of DEGs in RNA-seq data. *MEIS1* is the most highly DEG in this dataset (logFC = 5.15, FDR = 2.60E-16).

SK-N-SH cells overexpressing *MEIS1* (*MEIS1-*OE) in a stable manner were first characterized by western blot immunodetection (Fig 1A). The apparent molecular weight of MEIS1 is 43 kDa and SK-N-SH cells overexpressing it show a markedly higher level of MEIS1 by comparison to their control parental SK-N-SH cells. Overexpression of *MEIS1* was subsequently confirmed at the RNA level when the RNA-Seq data was analyzed; a Volcano plot shows *MEIS1* to be the most differentially expressed gene (DEG, log fold change (logFC) = 5.15, false discovery rate (FDR) = 2.60E-16, Fig 1B).

### Differential expression as a result of *MEIS1* overexpression in SK-N-SH cells

**Fig 2:**
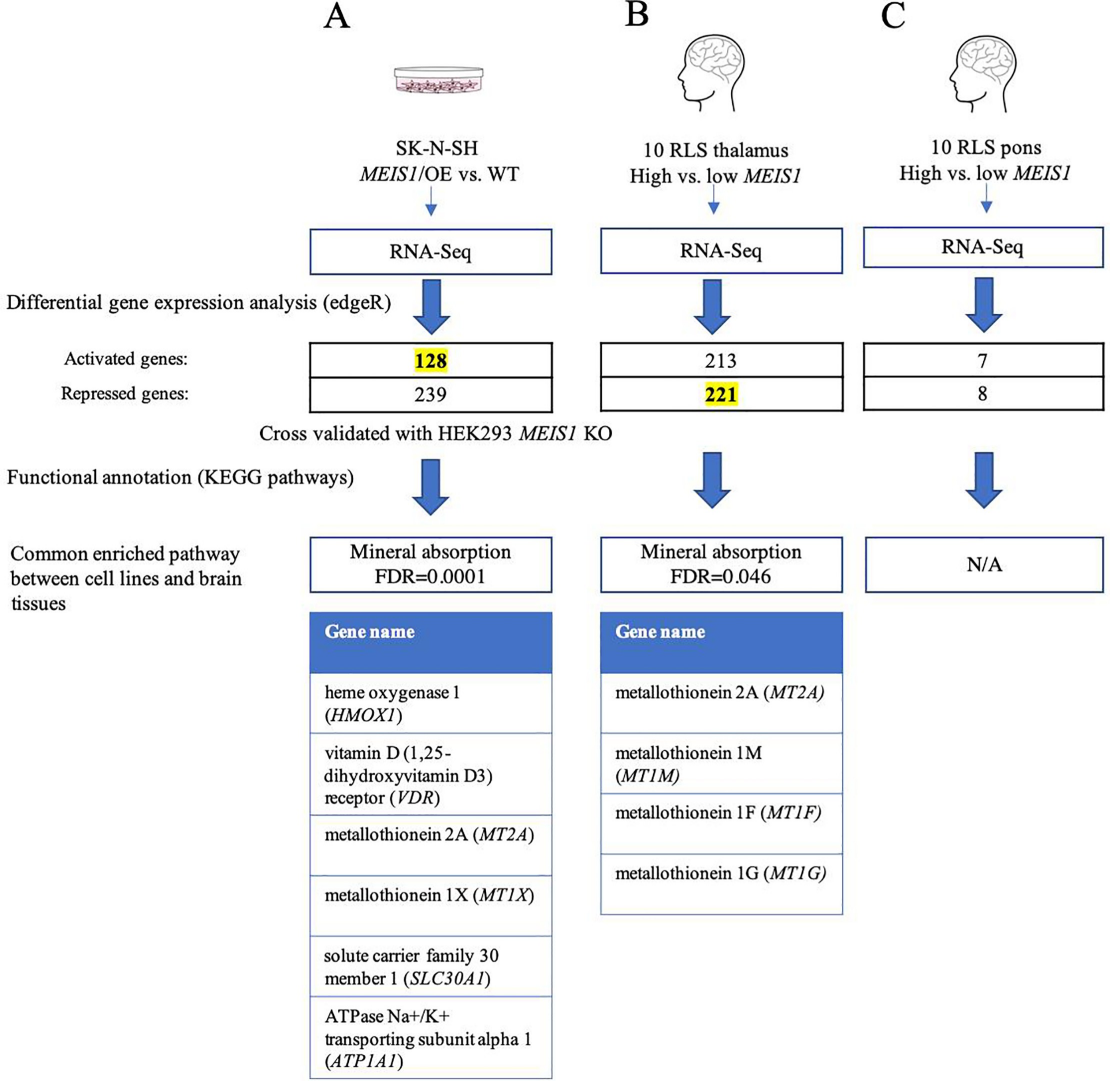
Schematic diagram representing the study design. Mineral absorption was enriched in the datasets highlighted in yellow (128 genes activated in cell lines, 221 repressed in thalamus).

A differential gene expression (DGE) analysis was made by comparing the RNA-Seq data obtained from *MEIS1*-OE cells to the wild-type SK-N-SH control. Overall 1,374 genes were observed to be significantly up-regulated, and 937 to be significantly down-regulated (FDR≤0.05). To further validate the differentially expressed genes and refine the data to a smaller list of the most significantly DEGs, we also used a human HEK293 cell line to knockout *MEIS1* and performed RNA-Seq experiment (S1 Text). Only the DEGs that were also differentially expressed and replicated in the KO cells were selected for further analysis, that involved functional annotation and validation in the brains of patients. As a result, 128 genes were activated and 239 genes were repressed (Fig 2A, S1 Table and S1 Text). Furthermore, our group has previously reported a reduced expression of another RLS associated gene, *SKOR1* in the thalamus region of RLS patients carrying the *MEIS1* risk haplotype with reduced *MEIS1* expression [18, 21]. The DGE analysis made in the current study using human cell lines showed that *SKOR1* is differentially expressed in SK-N-SH cells with a p-value of 0.0092; however, this result was not significant after correction for multiple testing (FDR = 0.082). This may suggest some limitations for using *in vitro* models to study neurological disorders. Nevertheless, *SKOR1* was not either a MEIS1 downstream gene in the RLS pons samples in the study by Catoire *et*. *al*. 2018 [18]; this information implicates that the regulatory role of MEIS1 as a transcription factor might vary in different conditions and the downstream genes found by *in vitro* studies in human cell lines are best to be confirmed in different brain regions related to RLS.

### *MYT1* is a differentially expressed gene previously associated with RLS

Considering that our team previously reported that *MEIS1* positively regulates the expression of *SKOR1* [18], we looked if MEIS1 regulated the expression of additional transcription factors. The RNA-Seq data derived from our cell line revealed the expression of myelin transcription factor 1 (*MYT1* gene) to be negatively regulated by MEIS1. *MYT1* is expressed in the neuronal progenitor cells and is involved in neuronal differentiation [22]. Interestingly a meta-analysis of RLS cases with European ancestry recently revealed an association between RLS and *MYT1* (p-value = 3.36E-14, OR = 1.13 95%CI 1.08–1.17) [12].

### DEGs activated by MEIS1 are enriched in caudate nucleus, a brain region with low iron levels in RLS patients

Information regarding the tissue expression of the 128 DEGs that are activated and the 239 DEGs that are repressed in the *MEIS1* dysregulated cells was obtained from the GNF SymAtlas gene expression atlas [23]. Tissues with enrichments of the DEGs are listed in Table 1. Interestingly, caudate nucleus (a component of basal ganglia) was the tissue showing the most significant enrichment (FDR = 7.40E-09) for DEGs that are activated in this dataset; 61 out of the 128 DEGs are expressed in caudate nucleus. The most consistent biological abnormality observed in RLS patients is brain iron deficiency within the substantia nigra, red nucleus, putamen, caudate nucleus and thalamus [24, 25]. This observation, in co-occurrence with the well-established iron deficiency that is observed in this brain region of RLS patients examined by MRI, emphasizes on the links that were previously made between MEIS1 and iron pathway in RLS [26].

**Table 1:** Tissue enrichment of the DEGs activated in *MEIS1* dysregulated human cell lines. Caudate nucleus is the tissue with the most significant enrichment of the DEGs.

Category	Term	p-value	FDR
**GNF_U133A_QUARTILE**	Caudatenucleus_3rd	9.40E-11	7.40E-09
**GNF_U133A_QUARTILE**	Heart_3rd	1.80E-07	6.90E-06
**GNF_U133A_QUARTILE**	Ovary_3rd	1.00E-04	1.90E-03
**GNF_U133A_QUARTILE**	Adrenal Cortex_3rd	9.10E-05	2.40E-03
**GNF_U133A_QUARTILE**	Atrioventricular node_3rd	5.10E-04	7.90E-03
**GNF_U133A_QUARTILE**	Pancreas_3rd	1.20E-03	1.30E-02
**GNF_U133A_QUARTILE**	Trachea_3rd	1.20E-03	1.50E-02
**GNF_U133A_QUARTILE**	Medulla Oblongata_3rd	2.20E-03	2.10E-02

### DEGs identified in human cell lines reveal mineral absorption to be a significantly enriched pathway

Enrichr and DAVID are two bioinformatics tools which provide functional annotation of large gene lists adopted from high throughput sequencing data [27–30]. We used Enrichr in conjunction with the KEGG database for the functional analysis of the DEGs and subsequently confirmed the results using DAVID. The only pathway that was significantly enriched in the DEGs lists after correction for multiple testing was mineral absorption, with MEIS1 having an activator function (FDR = 0.0001, Table 2). Here this enriched pathway includes *HMOX1*, *VDR*, *MT2A*, *MT1X*, *ATP1A1* and *SLC30A1* genes (Fig 2A). *HMOX1* (heme oxygenase 1) is required for the degradation of heme to biliverdin, free iron, and carbon monoxide and shows strong links to RLS pathways as it is involved in iron metabolism. This gene has previously been reported to be associated with RLS in a cohort of Spanish cases [31]. *VDR* (Vitamin D receptor) is another gene present in the mineral absorption pathway, and an association between *VDR* and RLS was also reported in Spanish cases [32]. Moreover, several studies have reported vitamin D to play an important role in RLS [17, 33, 34]. These previously unreported links between *MEIS1* and *HMOX1*, and between *MEIS1* and *VDR*, substantiate how the transcriptional regulatory role of MEIS1 might contribute to the pathogenicity of RLS. *MT2A* and *MT1X* are two metallothionein genes also present in this enriched pathway. Metallothioneins (MTs) belong to a family of proteins that bind intracellular metals and also have a protective role against free radicals generated by oxidative stress [35]. To predict whether MEIS1 regulatory role over these genes are a result of its direct binding to their promoter regions, we used the Meis1 chromatin immunoprecipitation sequencing (ChIP-Seq) data in mice available from GEO database (GSE82314) by Mahe *et al*. [36]. Promoter regions of *VDR* and *ATP1A1* orthologs in mice showed peaks in this ChIP-Seq data, raising the possibility that MEIS1 might directly regulate these genes by binding to their promoter sequences (S1 Text).

**Table 2:** The enriched pathway in the DEGs activated in *MEIS1* dysregulated human cell lines.

Term	p-value	FDR	Genes
**Mineral absorption**	8.69E-07	1.09E-04	*MT2A*, *VDR*, *HMOX1*, *SLC30A1*, *MT1X*, *ATP1A1*

### Differential gene expression in brains of RLS patients with contrasting levels of *MEIS1* expression

To build on an earlier report where we observed a reduced *MEIS1* expression level in cells and thalamus tissues derived from a subset of RLS patients carrying the *MEIS1* risk haplotype (GG/GG), [20] we performed an RNA-Seq experiment using tissues from the same brain regions of RLS patients. RNA-Seq data was generated from ten thalamus samples showing highly contrasting levels of *MEIS1* expression; five samples with substantially lower levels of *MEIS1* expression (who carry *MEIS1* risk haplotype, GG/GG) by comparison to five samples with higher expression levels (non-risk, AA/TT or heterozygous carriers, AG/TG) which was verified using a TaqMan gene expression assay (p-value<0.001, S2 Table). Pons tissues were also tested in that previous study, however *MEIS1* expression reduction was not significant as a result of *MEIS1* haplotype in this region. Nevertheless, we also used pons samples only based on their contrasting *MEIS1* expression to assess whether the previous observations would replicate. 434 genes were differentially expressed in thalamus and 15 genes were differentially expressed in pons comparing the high and low *MEIS1* expression cases (FDR≤0.05, S3 and S4 Tables). These two lists flag candidate genes that may be downstream to the transcription factor activity of MEIS1.

### Functional annotation of DEGs from thalamus shows mineral absorption to be an enriched pathway

Enrichr was used in conjunction with the KEGG database for the functional analysis of DEGs possibly regulated by MEIS1 in RLS brain tissues. This analysis was performed separately for each tissue and in both directions of differential gene expression; i.e. genes up or down-regulated associated with lower *MEIS1* expression in these two brain regions (potentially repressed or activated by MEIS1). The results of this analysis also identified mineral absorption to be enriched in DEGs that were repressed in samples of contrasting *MEIS1* expression in thalamus (FDR = 0.046, Fig 2B, Table 3). Nevertheless, this enrichment was not observed in the pons sample (Fig 2C). This observation is similar to our previous report where only thalamus samples showed lower *MEIS1* expression as a result of *MEIS1* risk haplotype [20]. The mineral absorption pathway was the only shared pathway across the functional analyses made from data derived from both the human cell lines and brain tissues. Metallothioneins are a common and prominent family of proteins in this pathway. It appears that in thalamus, MEIS1 may have a repressor role over the genes linked to this pathway (contrary to what was observed using human cell lines). This opposite direction might be explained by different mediatory pathways or genes that are present in cultured cells by comparison to the more complex nature of the brain tissue.

**Table 3:** Enriched pathways in DEGs repressed in thalamus samples with contrasting *MEIS1* expression.

Term	Overlap	p-value	FDR	Genes
**Protein processing in endoplasmic reticulum**	10/165	5.95E-06	8.45E-04	*DNAJA1*, *DNAJB1*, *HSP90AA1*, *HSPH1*, *HSPA1L*, *HSPA4L*, *HSPA6*, *TRAF2*, *HSPA1B*, *HSPA1A*
**Antigen processing and presentation**	6/77	1.15E-04	8.19E-03	*HSP90AA1*, *HSPA1L*, *HSPA4*, *HSPA6*, *HSPA1B*, *HSPA1A*
**Legionellosis**	5/55	2.10E-04	9.96E-03	*HSPA1L*, *HSPA6*, *HSPA1B*, *HSPD1*, *HSPA1A*
**Estrogen signaling pathway**	7/137	4.36E-04	1.55E-02	*HSP90AA1*, *HSPA1L*, *HSPA6*, *FKBP4*, *EGFR*, *HSPA1B*, *HSPA1A*
**Mineral absorption**	4/51	1.62E-03	4.60E-02	*MT2A*, *MT1M*, *MT1F*, *MT1G*

### Overlaps between DEGs identified in human cell lines and brain tissues with dysregulated *MEIS1* expression

**Fig 3:**
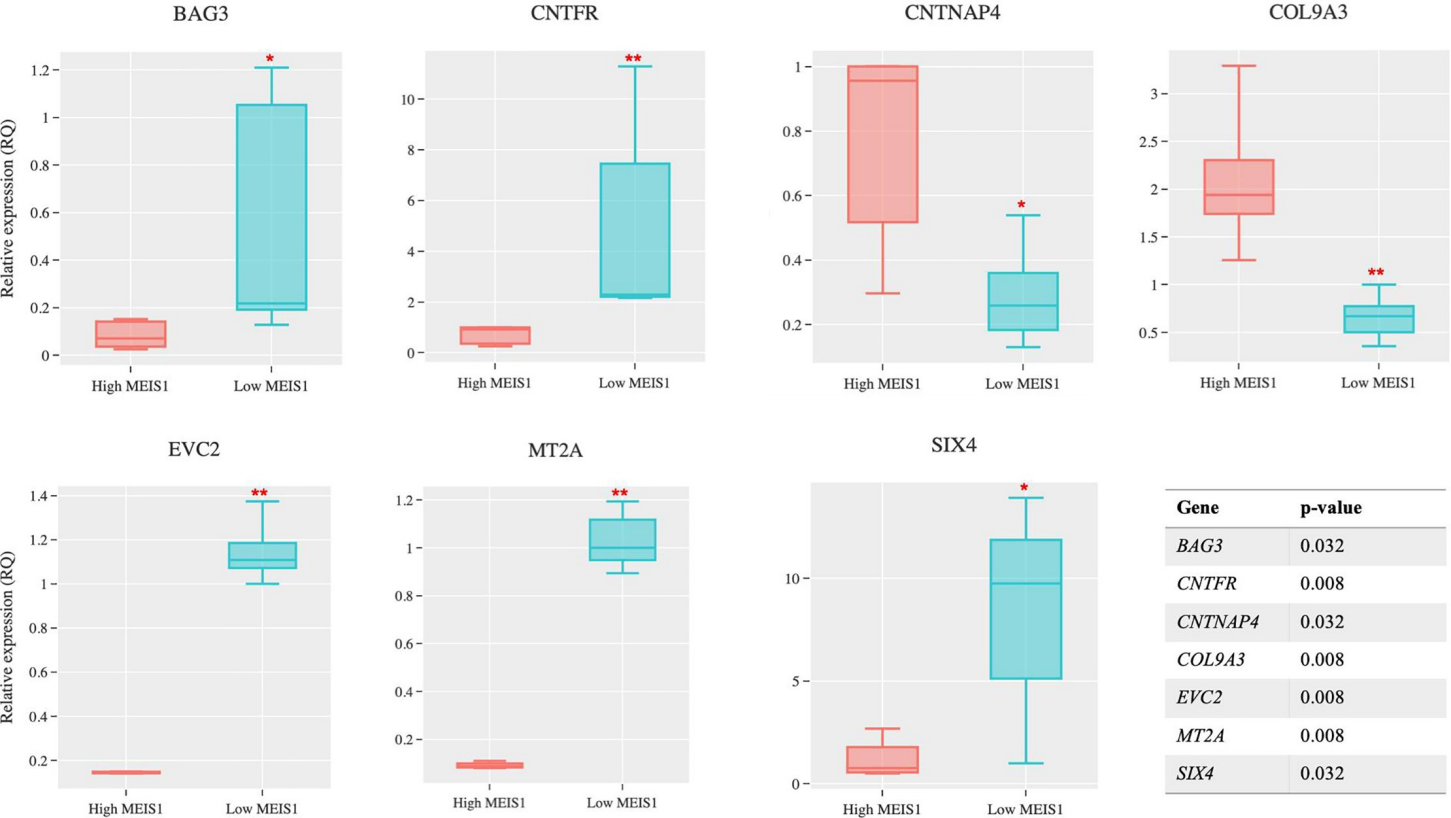
Expression Validation of the DEGs common between cell lines and thalamus by q-RT-PCR and their corresponding p-values. The expression levels of the seven DEGs present in both human cell lines and thalamus (as listed in Table 4) were measured by q-RT-PCR and their differential expression was assessed by non-parametric Wilcoxon test. **p≤0.01; *p≤0.05.

None of the 15 DEGs in pons overlapped with those identified using human cell models. Among the DEGs identified in thalamus samples, a list of seven DEGs overlapped with those identified using human cell models (Table 4) and their differential expression was also validated by quantitative reverse transcriptase PCR (q-RT-PCR, Fig 3). *CNTNAP4* which encodes a protein involved in dopaminergic synaptic transmission [37] and *CNTFR* which encodes a receptor for ciliary neurotrophic factor that is involved in neuronal survival are present in this list [38]. Among these seven genes, *MT2A* is the only gene in the mineral absorption pathway and a member of metallothionein protein family. We also performed q-RT-PCR on all the genes present in the mineral absorption pathway obtain from human cell lines (Table 2), to consider for the possibility of the brain tissues’ RINs affecting the ability of RNA-Seq in identifying all the DEGs; however, none of the genes present in the mineral absorption pathway (except *MT2A*) showed differential expression in thalamus (confirming the results obtained from RNA-Seq, S1 Text).

**Table 4:** The overlaps between the DEGs in thalamus and human cell lines with *MEIS1* dysregulation.

Gene	logFC	p-value	FDR
*BAG3*	2.91	4.42E-13	4.02E-09
*CNTFR*	1.67	1.09E-06	7.99E-04
*CNTNAP4*	-1.45	2.92E-05	8.65E-03
*COL9A3*	-2.025	5.83E-06	2.835E-03
*EVC2*	1.60	2.29E-04	3.735E-02
*MT2A*	1.31	3.79E-06	2.05E-03
*SIX4*	2.62	2.21E-08	4.01E-05

The presence of MEIS1 binding sites in the promoter regions of these seven genes common between human cell lines and thalamus samples was also assessed using the publicly available Meis1 ChIP-Seq data in mice (GEO database, GSE82314) [36]. Orthologs of *BAG3*, *CNTNAP4* and *EVC2* showed peaks in this ChIP-Seq data that suggest the possibility of a direct regulatory function for MEIS1 on these genes (S1 Text); this direct binding needs to be further validated in human cell lines.

To identify the neuronal specific MEIS1 targets, we also searched for the overlaps between the DEGs present only in SK-N-SH cells and the RLS brain tissues (SK-N-SH is a neuroblastoma cell line and may be superior to HEK293 for identification of the neuronal specific MEIS1 targets); 40 genes were common between these two conditions (S5 Table). Again, *MT2A* was the only gene present in the mineral absorption pathway and interestingly *NR1D1* was identified as a common DEG. *NR1D1* is a transcription factor that negatively regulates the core clock proteins and has a heme-dependent regulation of circadian rhythm [39, 40]. The links between *NR1D1* to iron metabolism, circadian rhythm and MEIS1 make this gene highly interesting in the RLS follow up functional studies.

## Discussion

In this study we used RNA-Seq to investigate the transcriptional regulatory function of MEIS1 in RLS. The *MEIS1* locus harbors the most significant genetic risk factor identified for RLS [12]. However, its role in RLS is yet to be identified. MEIS1 is a member of the homeobox containing transcription factor family, a class of proteins that has regulatory activities in a range of tissues. Following the identification of a *MEIS1* risk haplotype for RLS, our team examined the expression of this gene in material derived from patients (lymphoblastoid cells, thalamus and pons) and reported that the risk haplotype was associated with its reduced expression in LCL and thalamus region [20]. We subsequently used a *C*. *elegans* model to establish that *MEIS1* orthologue (*Unc-62*) is a regulator of proteins involved in iron homeostasis [26]. Finally, we recently established *MEIS1* to positively regulate the expression of another RLS associated gene, *SKOR1*, by directly binding to its promoter sequence [18, 21]. Common variants within *SKOR1* non-coding regions have been found to be associated with RLS and were replicated in several independent studies [12]. *SKOR1* is highly expressed in the central nervous system (CNS) of human and mouse and acts as a corepressor for a transcription factor called Lbx1 in mouse (*SKOR1* was previously called *LBXCOR1*). These two genes cooperatively act in regulation of cell fate in the dorsal horn interneurons of the spinal cord [41].

To build on these previous studies, we generated stable human cell lines in which *MEIS1* gene was either overexpressed or inactivated. Cross validation of the DEGs across these two conditions resulted in lists of 128 genes possibly activated and 239 genes possibly repressed by MEIS1. Functional annotation showed that mineral absorption is the only enriched pathway across the 128 activated DEGs. Six genes are present in this enriched pathway, among them *HMOX1* and *VDR* play roles in iron metabolism and response to vitamin D, respectively which are highly relevant to RLS. *MT2A* and *MT1X* are members of metallothionein family of proteins. To our knowledge, the regulatory role of MEIS1 over these genes is reported for the first time in this article.

Following the identification of DEGs downstream MEIS1 in human cell models, we generated RNA-Seq data from the thalamus and pons of RLS patients. These brain regions have been used in our earlier study, investigating the effects of *MEIS1* risk haplotype on its expression. We reported that thalamus samples with the risk haplotype showed a significantly lower *MEIS1* expression (this reduction was not significant in pons, though) [20]. We took advantage of the naturally occurring differential expression of *MEIS1* in brain tissues to validate DEGs and enriched pathways identified in the cell models. Functional annotation of the DEGs identified in brain tissues confirmed mineral absorption to be enriched in the DEGs repressed in thalamus samples with dysregulated *MEIS1* expression. Interestingly metallothioneins (MTs) are shared between the cell models and the brain tissues. MTs are small proteins that bind to intracellular metals [35]. Among the five types of MTs, the expression of types 1 and 2 is localized in the spinal cord and brain, where they regulate the cellular homeostasis of essential metals and protect cells from free radicals generated by the oxidative stress, type 3 metallothioneins have been mainly found in the neurons [35]. In Parkinson’s disease, low levels of antioxidants and high levels of free iron make grey matter vulnerable to reactive oxygen species (ROS) attack and it has been proposed that MTs (more specifically MT2A) released from astrocytes may play a role in protecting dopaminergic neurons from oxidative stress damage [35, 42]. It is also notable that previous studies have established interactions between metallothionein complexes and ferritin to trigger the simultaneous release of iron and zinc [43]. In rats it has been observed that intracellular iron deficiency results in higher expression of type 1 metallothionein [44]. The direction of MEIS1 regulatory function on MTs (and other genes in the mineral absorption pathway) was positive activation in the cell lines but negative repression in the brain. This divergence might be due to the fact that the pathways at play in the homogeneous cell lines will be far less numerous and complex than those of brain tissues which encompass multiple cell types interacting with one another. Considering that RLS underlying pathways are far from being completely understood and that the definition of its biology is poor in the literature so far, we believe that the enrichment of mineral absorption pathway that results from *MEIS1* dysregulation must receive more attention. This enrichment is consistent in two different cell types and in an RLS related brain tissue and is highly relevant to the most well know RLS pathway (iron metabolism). Future investigations are needed to identify the exact role of this pathway in RLS; such studies might reveal which mediators contribute to the divergent observations made in our cell models and brain tissues. One possible approach could be the use of single cell RNA sequencing (scRNA-Seq). Given the complexity of the brain tissues which are comprised of different cell types, scRNA-Seq can identify DEGs in different clusters of cell populations.

Another finding that arises from our cross validated RNA-Seq study, regardless of the enriched pathway, is the presence of seven thalamus DEGs in the cell line transcriptome. Three of the genes that are related to RLS pathways are the following: *CNTNAP4* (dopaminergic synaptic transmission) [37], *CNTFR* (a receptor of ciliary neurotrophic factor involved in neurons survival and differentiation) [38], and *MT2A* (an MT that is protective of oxidative stress [35] and is linked to renal aging processes [45]). These RLS related pathways include dopaminergic transmissions, higher risk of RLS in patients with chronic kidney disease and also neurodevelopmental basis of RLS [46, 47].

Overall, the regulatory connection of *MEIS1* to RLS pathways was studied in four different settings, i.e. two cell lines and two brain regions. A comparison of the DEGs identified in these settings revealed that the enrichment of MT genes from the mineral absorption pathway is common to cells and thalamus. Further future studies will be required to unravel intricate deleterious implications that might be associated with the changes in MTs expression levels and RLS.

## Methods

### Generation and characterization of the human neuroblastoma cell lines overexpressing *MEIS1* gene

The cDNA of *MEIS1* (NM_002398.2) was sub-cloned in pcDNA3.1+ (at BamH1 and EcoR1 restriction cloning sites), a mammalian expression vector with strong CMV promoter which contains a neomycin selection marker (Open Biosystems, clone ID: 5531644). The cDNA of the plasmid was sent for Sanger sequencing to validate the sequence. Human SK-N-SH cells were transfected with this vector using jetPRIME® (Polyplus). G418 antibiotic was used to select stable cell lines overexpressing *MEIS1* (a standard curve established the optimum G418 concentration for SK-N-SH cells to be 500 ug/ml). 48 hours post transfection, the cells were transferred to DMEM containing 500 ug/ml G418 and the media was changed every other day for a period of four weeks to obtain stable cell lines overexpressing *MEIS1*. The case and the control conditions were done in at least triplicates. Characterization of MEIS1 overexpression was performed by western blot analysis using polyclonal anti-MEIS1 from Abnova (Catalog number: H00004211-A01, http://www.abnova.com).

### RNA extraction from human cell lines

Human cell line total RNA was extracted using RNeasy® mini kit (Qiagen). The RNA concentration was measured using the Synergy H4 Hybrid Multi-Mode Microplate reader from BioTek and 250 ng RNA in 25 ul of nuclease free water was provided to the McGill University and Génome Québec Innovation Centre. RNA Integrity Number (RIN) was assessed using the 2100 Bioanalyzer instrument, together with the 2100 Expert software and Bioanalyzer assays (RIN > 9 for all the cell line RNA samples). Eukaryotic long noncoding and coding transcripts (rRNA-depleted libraries) were targeted for sequencing using Illumina Ribo-Zero rRNA Removal Kit (Human/Mouse/Rat).

### RNA extraction from human brain tissues

Brain tissues (ten thalamus and ten upper pons) were obtained from autopsy brain tissues of individuals (Caucasians of European ancestry) with an RLS diagnosis from the Harvard Brain Tissue Resource Centre. Final diagnosis was made by an RLS expert physician based on available questionnaires and medical records but blinded to the genotype information. Total RNA was extracted from 0.2 g of frozen brain tissue using the RNeasy^®^ Lipid Tissue kit (Qiagen). Throughout the RNA extraction a randomization process was used to ensure that no batch effects were generated. The RNA concentration was measured using the Synergy H4 Hybrid Multi-Mode Microplate reader from BioTek and 2.5ug RNA in 50 ul of nuclease free water was provided to Macrogenlab Inc. RINs were assessed using the 2100 Bioanalyzer instrument, together with the 2100 Expert software and Bioanalyzer assays (S2 Table). Higher RINs are required for library preparations with Ribo-Zero method, since degraded RNA samples can be removed during this procedure and this can introduce a bias in the results. Poly (A) selection method can also introduce a strong 3′bias [48]. Therefore, Ribo-Zero or poly (A) selection methods may not be optimal for our degraded but highly valuable brain RNA samples. Alternatively, RNA access [49] is the method of choice that produces high-quality data from degraded RNA samples from high-value content regions (most specifically in the brain). Brain RNA samples for both thalamus and pons were matched for post mortem interval (PMI), age, sex and RNA integrity number, and there were no group differences in these variables (S2 Table).

### High throughput transcriptome sequencing

RNA sequencing [50] was done on the Illumina HiSeq 2500 platform at 100bp paired-end reads with a total of 80 million reads per sample (the cell line transcriptomes were sequenced at McGill University and Génome Québec Innovation Centre and the post mortem brain tissue RNA samples were sequenced at Macrogenlab Inc).

### Bioinformatic analyses

Following high throughput sequencing, the FASTQ files of the paired-end reads were aligned to the human genome reference (GRCh37/hg19 assembly) using STAR v2.5.1b [51]. Picard v1.123 was used to mark duplicates, calculate exonic/intronic/intergenic rates and generate RNA metrics using both CollectRnaSeqMetrics and CollectAlignmentSummaryMetrics functions (https://broadinstitute.github.io/picard/). Gene-level quantification was done using HTSeq-count and all the genes with less than 1 read per sample were removed. (http://www-huber.embl.de/users/anders/HTSeq/doc/overview.html). Cufflink was also used to generate the gene-expression values as FPKMs (fragments per kilobase of exon per million fragments mapped) [52]. The library size for each sample was estimated using the number of mapped reads in the BAM file (ttp://samtools.sourceforge.net/). The above steps were run on GenPipes rnaseq.py v3.0.0 [53]. EdgeR v3.5.2 was used to determine differentially expressed genes (DEGs) [54]. Likelihood ratio test included age, post-mortem interval (PMI), RNA integrity number (RIN) and batches as covariates for the brain tissues in an additive model. Including RIN in this additive model can retrieve the biologically meaningful results from these low RIN but precious RNA samples [55]. An exact test with no covariate was used for cell line RNA-Seq data analysis. Gene annotations were incorporated using the biomaRt v2.14.0 R package (http://www.bioconductor.org/packages/release/bioc/html/biomaRt.html). The list of significantly differentially expressed genes (DEGs) was defined at FDR≤0.05. DAVID (v6.8) and Enrichr were used for the Gene Ontology (GO) and KEGG analysis [29, 30].

### Quantitative reverse transcriptase PCR (q-RT-PCR)

Single-stranded cDNA synthesis was performed from 1 μg of total RNA using the SuperScript® VILO^™^ cDNA Synthesis Kit (Invitrogen). Quantitative RT-PCR was performed using the TaqMan method (Applied Biosystems) with probes and primers designed by Applied Biosystems for *MEIS1* (Hs00180020-m1), *MT2A* (Hs02379661_g1), *CNTFR* (Hs00181798_m1), *CNTNAP4* (Hs00369159_m1), *VDR* (Hs01045843_m1), *HMOX1* (Hs01110250_m1), *SLC30A1 (Hs00253602_m1)*, *MT1X* (Hs00745167_sH), *ATP1A1* (Hs00167556_m1), *SIX4* (Hs00213614_m1), *COL9A3* (Hs00951243_m1), *BAG3* (Hs00188713_m1), *EVC2* (Hs00377633_m1). PCR conditions were as follows: 50°C for 2 min, 95°C for 10 min, followed by 40 cycles at 95°C for 15 sec (denaturation) and 60°C for 1 min (annealing and extension). Fluorescent signals were captured using the QuantStudio^™^ 7 Flex Real-Time PCR System and Software (v1.0) (Applied Biosystems). The level of expression was determined by converting the threshold cycle (Ct) values using the 2^-ΔΔCt^ method. Expression levels were normalized using the human *POLR2A* control (Hs00172187_m1). All experiments were run in triplicate. Independent cDNA synthesis was carried out twice.

### Statistics

Statistics tests were conducted in R v3.5.1 (http://R-project.org/).

### Study approval

RLS brain tissues were provided by the Harvard Brain Tissue Resource Center, which is supported in part by a Public Health Service Grant (R24MH068855), with permission from the RLS Brain Bank Tissue Review Committee through the RLS Foundation. This study was approved by Comité d’éthique de la recherche du Centre hospitalier de l′Université de Montréal and McGill University ethics, all methods were performed in accordance with the relevant guidelines and regulations of McGill University (REB NEU-14-051).

## Supporting information

S1 Text (DOCX)

S1 raw images The raw western blot images corresponding to Fig 1A. (PDF)

S1 Table Human cell lines DEGs. (XLSX)

S2 Table Demographics for RNA-seq study. (XLSX)

S3 Table Thalamus DEGs. (XLSX)

S4 Table Pons DEGs. (XLSX)

S5 Table DEGs Thalamus SK-N-SH. (XLSX)

S6 Table DEGs SK-N-SH. (XLSX)

S7 Table DEGs HEK293. (XLSX)
