# Supplementary material for: Mineral absorption is an enriched pathway in a brain region of restless legs syndrome patients with reduced MEIS1 expression
Source: PLoS One. 2019 Nov 14;14(11):e0225186. doi: 10.1371/journal.pone.0225186 (PMC6855629; doi:10.1371/journal.pone.0225186)
Supplement: S1 Text — (DOCX) [file pone.0225186.s001.docx]

# **S1 Text**

# **Supplemenal data**

## ***MEIS1* single guide RNA design and cloning for the generation of *MEIS1* knockout cells by CRISPR-Cas9**

Single guide RNA (sgRNA) sequences targeting the second exon of *MEIS1* gene (to introduce indels, resulting in a frameshift) were picked using the online CRISPR design tool from Zhang lab at the Broad institute (<http://crispr.mit.edu>), and were predicted to have a low probability of off-target sites. The most efficient sgRNA based on the T7 endonuclease assay was selected [1]. This sgRNA was then cloned into pSpCas9 (BB)-2A-Puro (PX459) vector (obtained from Dr. Feng Zhang, Addgene plasmid #48139) [2].

## **CRISPR/Cas9 mediated *MEIS1* knockout**

HEK293 cells were cultured in DMEM media and were transfected with the PX459 plasmid using jetPRIME® (Polyplus). 48 hours post transfection the DMEM media was replaced with media containing 2.5ug/ml puromycine (optimized concentration using a standard curve). 51 single colonies were isolated after four weeks and based on gene specific PCR 17 colonies were positively targeted. Three positive clones were chosen to precede the experiment in triplicates.

## ***MEIS1* knockout (*MEIS1*-KO) validation in HEK293 cells**

Sanger sequencing of the CRISPR-Cas9 targeted genomic region of *MEIS1* (S1 Fig) revealed a 47bp insertion plus 2bp deletion that led to a premature stop codon in clone 151, a 208bp deletion in clone 282, and an 11bp deletion in clone 461 (SnapGene software, S2A Fig) on one allele [2]. Moreover, the IGV [3, 4] view of the RNA-seq data alignment obtained from *MEIS1*-KO cells revealed the edition on the second allele. This showed the *MEIS1* RNA sequence to be disrupted at the CRISPR-Cas9 cut site for which the sgRNA was designed (3 bp upstream the pam sequence, S2B Fig). The *MEIS1* sgRNA led to a 2 bp deletion in clone 151 and 1bp insertion in clones 282 and 461, thus frameshifting the ORF of the canonical isoform which shows the edit on the second allele. Gene specific RT-PCR of the targeted region of *MEIS1* gene by CRISPR-Cas9, showed no mRNA band in the KO cells for the specific fragment length of 855bp, for the disruption of primer binding site in the region (S3A Fig). Moreover, western blot analysis showed two bands for the wild-type control cells versus a single band for the KO cells at MEIS1 molecular size (~43 kDa), suggesting no expression of MEIS1 protein in the KO cells (S3B Fig).


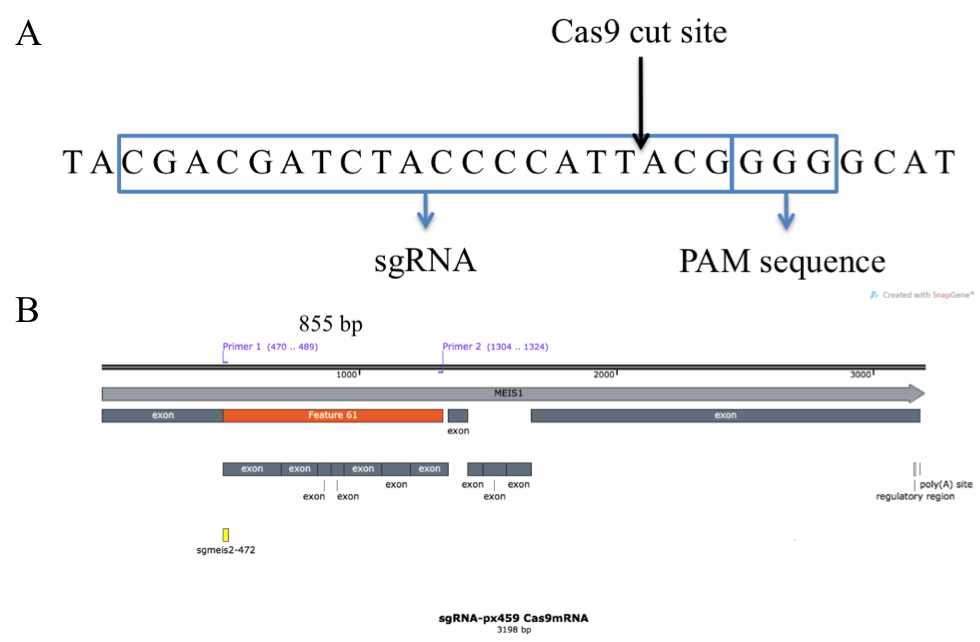


**S1 Fig. A.** sgRNA sequence for *MEIS1* gene, exon 2. **B.** The location of the sgRNA (yellow box) in *MEIS1* gene. The forward and reverse primers for gene specific RT-PCR surround the CRISPR targeted region.


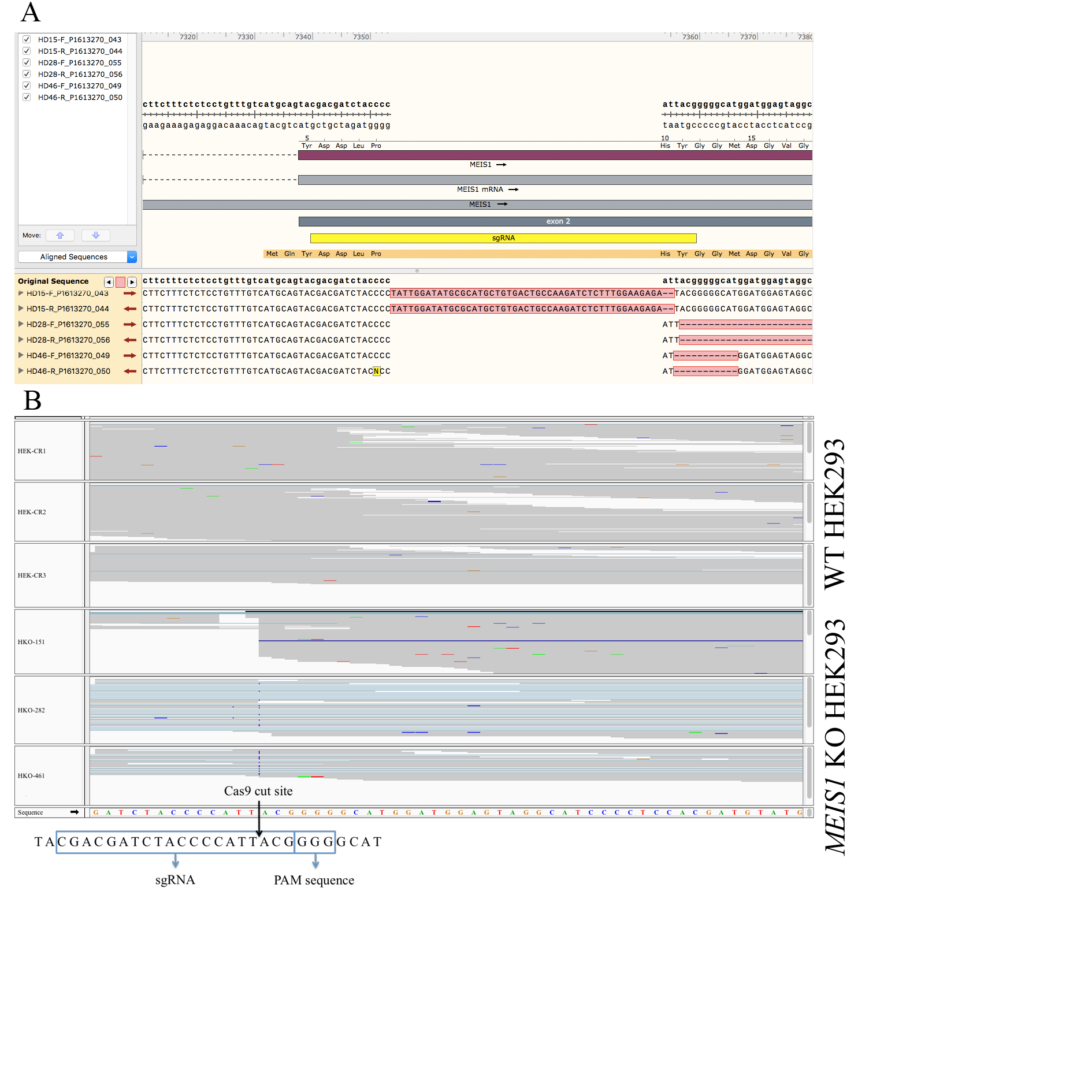


**S2 Fig. Characterization of *MEIS1* KO cells.** **A.** Sanger sequencing of the KO cells shows insersions in clone 151 and deletions in clone 282 and 461 on one allele. (SnapGene software) **B.** IGV view of the alignment of RNA-seq reads to *MEIS1* gene. The black arrow points the CRISPR/Cas9 cut site. 2bp deletion in clone 151 and 1pb insersion in clones 282 and 461 on the other allele at exact Cas9 cut site.


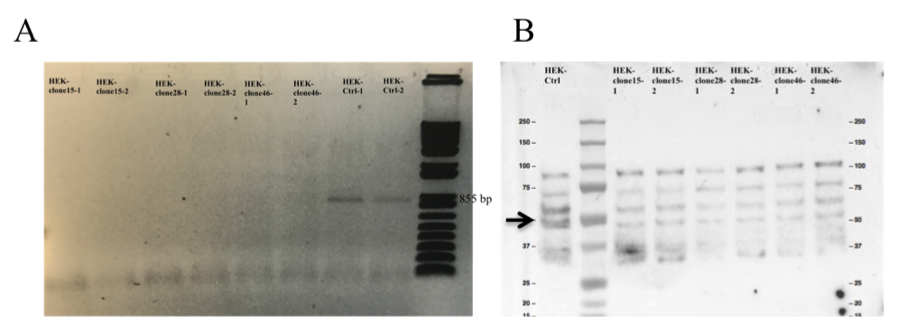


**S3 Fig. Characterization of *MEIS1* KO cells. A.** Gene specific RT-PCR resulting in no expression band in the *MEIS1* KO cells compared to control WT. **B.** Western blot analysis. The black arrow points the double band in the WT and the single band in the KO cells at the MEIS1 molecular size (43 kDa).

## **Cross validation of DEGs in *MEIS1*-OE neuroblastoma cells with *MEIS1-*KO HEK293 cells**

Differential gene expression analysis identified 5,606 DEGs that were significantly up-regulated in *MEIS1-*OE cells (1,374 DEGs) and *MEIS1*-KO cells (4,232 DEGs). Conversely 3,491 DEGs were significantly down-regulated in *MEIS1-*OE cells (937 DEGs) and *MEIS1*-KO cells (2,554 DEGs), available in S6 and S7 Tables. DEGs that were common across the *MEIS1*-KO and *MEIS1*-OE DEGs lists were selected for further investigations. Overall, 128 genes were observed to be activated by *MEIS1* as they were up-regulated in *MEIS1-*OE and down-regulated in *MEIS1*-KO cells. Conversely, a list of 239 repressed genes were observed to be down-regulated in *MEIS1-*OE cells while they were up-regulated in *MEIS1*-KO cells (S4 Fig).

A

B

**S4 Fig.** **Venn diagram of the DEGs in the *MEIS1* OE and KO cells and their overlapping genes**. **A.** 128 genes are possibly positively regulated (activated) by MEIS1. **B.** 239 genes are possibly negatively regulated (repressed) by MEIS1 in this dataset.

**Investigating the publicly available MEIS1 ChIP-Seq data**

Based on the fact that MEIS1 is a homeobox containing transcription factor and has direct DNA binding properties, we analyzed the chromatin immunoprecipitation sequencing (ChIP-Seq) data on Meis1 by Mahe *et al.* in 2017 (the experiment was performed in mice and the data is publicly available from GEO database, GSE82314) [5]. We searched for Meis1 DNA binding sites in the upstream ATG start site and the intronic region of the six genes in the cell line mineral absorption pathway (Table 2) and also the seven genes common between cell lines and thalamus (Table 4). The results are respectively presented in S5 and S6 Figs. Integrated genome browser (IGB) [6] was used for visualization; *Vdr, Atp1a1, Bag3, Cntnap4* and *Evc2* showed peaks in their upstream ATG start sites or their intron 1. This suggests that these genes are possibly directly bound by Meis1 and regulated by it. However, these binding regions are best to be assessed by specific protein-DNA binding assays on human cell models as well.

**S5 Fig.** IGB view of the Meis1 ChIP-Seq data (available from GEO database (GSE82314) by Mahe *et al*.) of genes present in the cell line mineral absorption pathway. **A.** Showing two peaks upstream *Vdr* (in orange). **B.** Showing two peaks upstream *Atp1a1* and one peak in its first intron (in orange).

**S6 Fig.** IGB view of the Meis1 ChIP-Seq data (available from GEO database (GSE82314) by Mahe *et al*.) of DEGs common between human cell lines and thalamus. **A.** Showing one peak in intron 1 of *Bag3* (in orange). **B.** Showing six peaks upstream *Cntnap4* (in orange). **C.** Showing one peak in intron 1 of *Evc2* (in orange).

**Quantitative reverse transcription PCR (q-RT-PCR) validation of DEGs present in cell line mineral absorption pathway**

The low RIN of RNA samples obtained from human brain tissues may result in some DEGs not being identified by RNA-Seq. Considering this possibility, we also performed q-RT-PCR to assess whether the DEGs present in the mineral absorption pathway resulted from cell line RNA-Seq, are differentially expressed in the thalamus or not (based on the RNA-Seq data, *MT2A* was the only gene in this pathway that was replicated in both cells and brain). The expression levels of these genes (Table 2) were measured by q-RT-PCR and their differential expression was analyzed by non-parametric Wilcoxon test. None of the genes showed a significant differential expression in the thalamus. This further validates the results obtained from brain tissues RNA-Seq (S7 Fig).

**
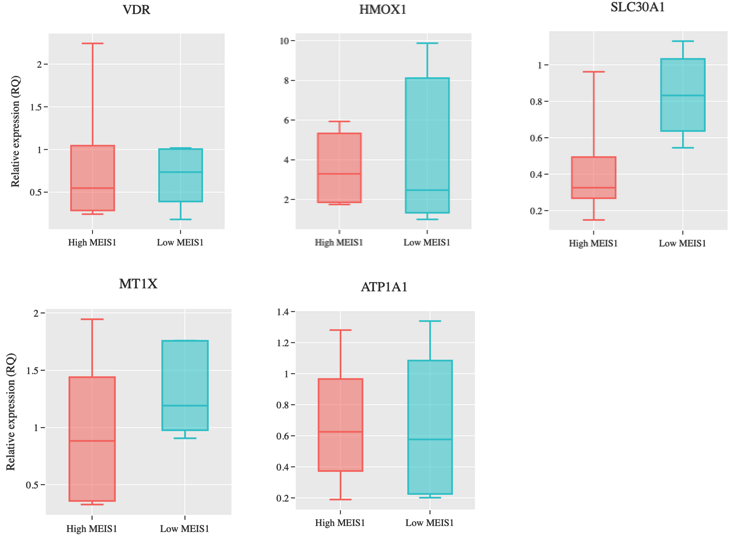
**

**S7 Fig.** q-RT-PCR examination of the human cell lines DEGs, present in the mineral absorption pathway in the thalamus samples with high vs. low *MEIS1* content. None of the genes showed a significant differential level of expression identified by q-RT-PCR (p-value>0.05).

**References.**

1. Vouillot L, Thelie A, Pollet N. Comparison of T7E1 and surveyor mismatch cleavage assays to detect mutations triggered by engineered nucleases. G3. 2015;5(3):407-15. doi: 10.1534/g3.114.015834. PubMed PMID: 25566793; PubMed Central PMCID: PMC4349094.

2. Ran FA, Hsu PD, Wright J, Agarwala V, Scott DA, Zhang F. Genome engineering using the CRISPR-Cas9 system. Nature protocols. 2013;8(11):2281-308. doi: 10.1038/nprot.2013.143. PubMed PMID: 24157548; PubMed Central PMCID: PMC3969860.

3. Robinson JT, Thorvaldsdottir H, Winckler W, Guttman M, Lander ES, Getz G, et al. Integrative genomics viewer. Nature biotechnology. 2011;29(1):24-6. doi: 10.1038/nbt.1754. PubMed PMID: 21221095; PubMed Central PMCID: PMC3346182.

4. Thorvaldsdottir H, Robinson JT, Mesirov JP. Integrative Genomics Viewer (IGV): high-performance genomics data visualization and exploration. Briefings in bioinformatics. 2013;14(2):178-92. doi: 10.1093/bib/bbs017. PubMed PMID: 22517427; PubMed Central PMCID: PMC3603213.

5. Mahe EA, Madigou T, Serandour AA, Bizot M, Avner S, Chalmel F, et al. Cytosine modifications modulate the chromatin architecture of transcriptional enhancers. Genome research. 2017;27(6):947-58. doi: 10.1101/gr.211466.116. PubMed PMID: 28396520; PubMed Central PMCID: PMC5453328.

6. Nicol JW, Helt GA, Blanchard SG, Jr., Raja A, Loraine AE. The Integrated Genome Browser: free software for distribution and exploration of genome-scale datasets. Bioinformatics. 2009;25(20):2730-1. doi: 10.1093/bioinformatics/btp472. PubMed PMID: 19654113; PubMed Central PMCID: PMC2759552.
