# Supplementary material for: Mineral absorption is an enriched pathway in a brain region of restless legs syndrome patients with reduced MEIS1 expression
Source: PLoS One. 2019 Nov 14;14(11):e0225186. doi: 10.1371/journal.pone.0225186 (PMC6855629; doi:10.1371/journal.pone.0225186)
Supplement: S1 raw images — (PDF) [file pone.0225186.s002.pdf]

Figure 1A was generated from the following raw blots. Chemiluminescence Western blot detection of the over-expressed proteins

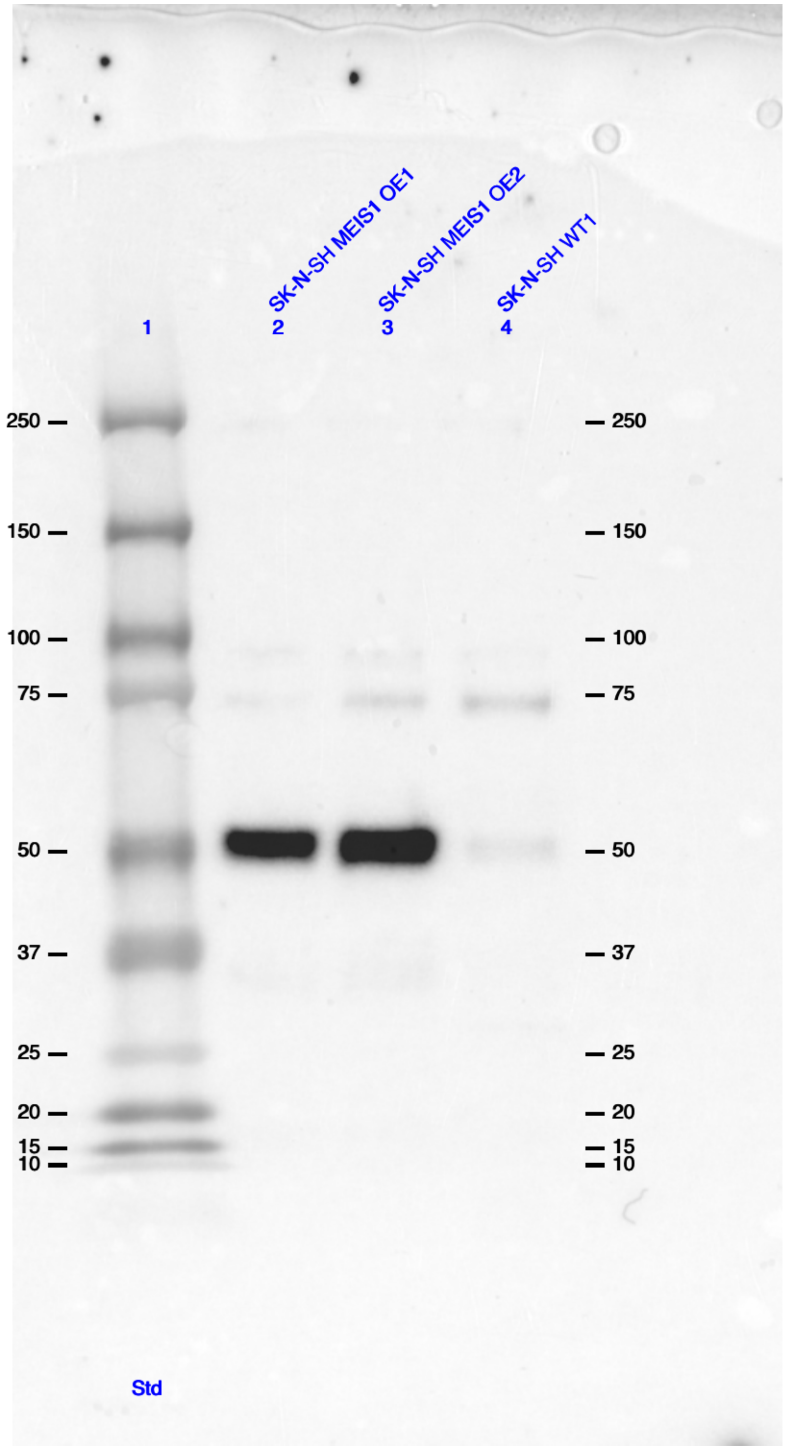

S8 Fig. Original western blot image corresponding Fig 1A

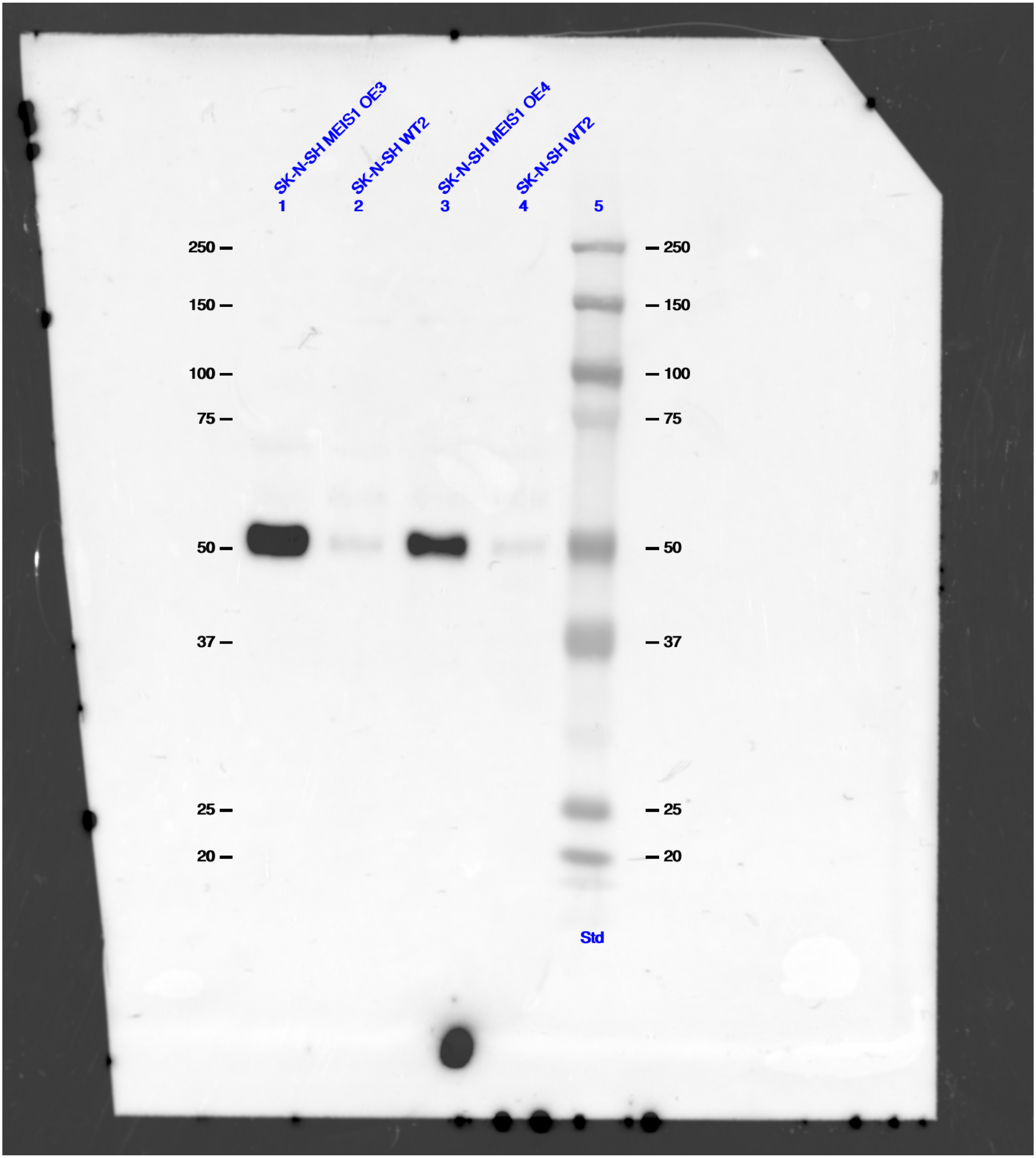

S9 Fig. Original western blot image corresponding Fig 1A
